# Persistent Basophilia May Suggest an “Accelerated Phase” in the Evolution of CALR-Positive Primary Myelofibrosis Toward Acute Myeloid Leukemia

**DOI:** 10.3389/fonc.2019.00872

**Published:** 2019-09-06

**Authors:** Jerome Dobrowolski, Sergiu Pasca, Patric Teodorescu, Cristina Selicean, Ioana Rus, Mihnea Zdrenghea, Anca Bojan, Adrian Trifa, Bogdan Fetica, Bobe Petrushev, Ana-Maria Rosu, Ioana Berindan-Neagoe, Ciprian Tomuleasa, Delia Dima

**Affiliations:** ^1^Department of Hematology, Iuliu Hatieganu University of Medicine and Pharmacy, Cluj Napoca, Romania; ^2^Department of Hematology, Research Center for Functional Genomics and Translational Medicine, Iuliu Hatieganu University of Medicine and Pharmacy, Cluj Napoca, Romania; ^3^Department of Hematology, Ion Chiricuta Clinical Cancer Center, Cluj Napoca, Romania; ^4^Department of Genetics, Ion Chiricuta Clinical Cancer Center, Cluj Napoca, Romania; ^5^Department of Pathology, Ion Chiricuta Clinical Cancer Center, Cluj Napoca, Romania; ^6^Department of Pathology, Regional Institute for Gastroenterology and Hepatology, Cluj Napoca, Romania; ^7^Research Center for Functional Genomics and Translational Medicine, Iuliu Hatieganu University of Medicine and Pharmacy, Cluj Napoca, Romania

**Keywords:** primary myelofibrosis, basophilia, leukemic transformation, accelerated phase, clinical prognosis

## Abstract

Basophils are white blood cells that play an important role in the human immune system. These cells physiologically increase in number in immune response to certain allergies, chronic inflammation, and parasitic infections. Basophils are also a significant indicator for the presence of certain malignancies such as chronic myeloproliferative neoplasms and acute myeloid leukemia. In the current manuscript we present a statistically significant correlation between persistent basophilia in primary myelofibrosis (PMF) and the risk for the subsequent development of acute myeloid leukemia. We have retrospectively identified in the files of the Department of Hematology, Ion Chiricuta Clinical Cancer Center in Cluj Napoca, Romania 623 consecutive patients diagnosed with AML over a period spanning from 2008 to 2018. We afterwards identified 32 patients with AML diagnosis following a previous diagnosis of myelofibrosis (either post-PV, post-ET, or post-PMF). All the patients were diagnosed according to the WHO criteria. We subsequently established a control group consisting of 32 patients with underlying BCR–ABL-negative MPN who did not develop AML (AML-negative group). Following this, we assessed whether the AML-negative patients from our control group also had a persistent (>3 months) absolute basophilia. When comparing both groups of patients with myelofibrosis, the group with subsequent AML development and the one without AML, the follow-up did not present statistically significant differences between the two groups. In the univariate analysis, patients who progressed to AML had more frequently basophilia, longer basophilia duration, higher pre-therapy absolute, and relative basophil count and presented more frequently calreticulin (CALR) mutations. In the current study, we emphasize the need for a closer clinical monitoring for chronic MPNs with marked basophilia, with an important potential clinical impact.

## Introduction

Peripheral blood basophilia is defined as a basophil count >0.1 × 10^9^/L and is rarely diagnosed. Still, when diagnosed, it may be caused by iron deficiency, hypersensitivity disorders, infections, or chronic inflammation (1, 2). Clinicians should always exclude these pre-existing conditions and should prompt the assessment of an underlying myeloproliferative neoplasm (MPN). MPNs include chronic myeloid leukemia (CML), polycythemia vera (PV), essential thrombocythemia (ET), primary myelofibrosis (PMF), and less common chronic neutrophilic leukemia, chronic eosinophilic leukemia not otherwise specified, MPN unclassifiable, and mastocytosis (3, 4). The molecular hallmark of CML is the *BCR-ABL* fusion, whereas for *BCR-ABL*-negative MPNs (PMF, PV, ET) common mutations include *JAK2* V617F and those within *CALR* exon 9. Basophilia may also be associated with other rare hematological malignancies, such as acute basophilic leukemia or acute myeloid leukemia with *t*_(6;9)_ (5, 6). Even if bone marrow morphology and cytogenetics are the backbone of diagnosing and monitoring a BCR-ABL-negative MPN, no current guidelines have yet to be published on the clinical algorithm in which a PMF with basophilia should be managed. Primary myelofibrosis is an MPN characterized by a progressive bone marrow failure syndrome with worsening cytopenia, with some of the patients later developing acute myeloid leukemia (AML) (7). Previously published data in patients with MPNs has shown that the development of monocytosis is associated with poor prognosis due to an “accelerated phase” of the disease (8). Thus, our hypothesis is that concomitant basophilia in PMF might be a hallmark for the development of such an “accelerated phase” and later AML development. We analyzed all the AML cases diagnosed following a PMF in the last 10 years in our institution and compared this cohort to a control group, consisting of primary diagnosed PMF without AML development. We have shown a correlation between persistent basophilia in CALR-positive and later AML diagnosis.

## Materials and Methods

### Case Selection

We retrospectively identified in the files of the Department of Hematology, Ion Chiricuta Clinical Cancer Center in Cluj Napoca, Romania 623 consecutive patients diagnosed with AML over a period spanning from 2008 to 2018. We afterwards identified 32 patients diagnosed with AML following a previous diagnosis of myelofibrosis (either post-PV, post-ET, or post-PMF). All the patients were diagnosed according to the WHO criteria. Peripheral blood counts were reviewed to identify patients with myelofibrosis, who at some point during their clinical history developed persistent absolute basophilia (>0.1 × 10^9^/L) for more than 3 months. We then investigated the clinical files of patients with basophilia and excluded those with any clinical condition known to be possibly associated with reactive basophilia. The excluded basophilia patients include those with chronic inflammation due to conditions such as infectious diseases, parasitic infections, inflammatory bowel diseases, and rheumatic arthritis. Our study also excluded patients with allergies, including food and drug allergies and allergic rhinitis.

To strengthen our hypothesis, we subsequently established a control group consisting of 32 patients with underlying PMF who did not develop AML (AML-negative group). Both cohorts, the MPNs that developed AML as well as the MPNs that did not develop subsequent AML, had the same population characteristics regarding sex distribution, median age, and follow-up time of 24 months. Following this, we assessed whether the AML-negative patients from our control group also displayed a persistent (>3 months) absolute basophilia. The median time to AML evolution was 18 months for the MPNs that developed an “accelerated phase AML” and then acute leukemia, with the evolution time ranging from 12 to 24 months. The design of this study was approved by the Institutional Review Board of Ion Chiricuta Oncology Institute in Cluj Napoca, Romania.

### Statistical Analysis

Data analysis and plot generation for the AML-positive cohort were executed using R version 3.5.1 (2018-07-02) (R Development core team, USA). Shapiro-Wilk and histogram visualization were used to determine the normality of data distribution. The distribution of all data was non-normal. Mann Whitney Wilcoxon test was used for comparing non-normally distributed continuous variables between two groups. A *p* value under 0.05 was considered statistically significant. Data analysis concerning the control group was performed using R 3.5.3. Fisher's exact test was used for analyzing contingency tables. The Shapiro–Wilk test and histogram visualization were used to determine the normality of distribution. Non-normal distributed data were represented as median (quartile 1, quartile 3). The Wilcoxon test was used to determine the difference between two groups with non-normally distributed variables. A random forest was used to rank the importance of the selected statistically significant variables. A *p* value under 0.05 was considered statistically significant.

Data analysis for the AML-negative cohort was performed using R 3.5.3. Fisher's exact test was used for analyzing contingency tables. The Shapiro–Wilk test and histogram visualization were used to determine the normality of distribution. Non normal distributed data were represented as median (quartile 1, quartile 3). The Wilcoxon test was used to determine the difference between two groups with non-normally distributed variables. A random forest was used to rank the importance of the selected statistically significant variables. A *p* value under 0.05 was considered statistically significant.

## Results

Subsequent to the diagnosis of myelofibrosis (either post-PV, post-ET, or PMF), 32 patients were diagnosed with AML. This cohort was composed of 19 men and 13 women with a mean age of 56.43 at diagnosis (range: 33–80). The duration was calculated by reviewing all available peripheral blood counts preceding and following the follow-up bone marrow biopsy to establish the time period during which basophilia remained sustained with no interruption. Following this, we performed an identical approach on the AML-negative control group. The control group was composed of 32 patients with a subsequent diagnosis of myelofibrosis (either post-PV, post-ET, or PMF) and no presence of acute myeloid leukemia. The group included 21 males and 11 females with a mean age of 61.65 at diagnosis (range: 17–83). The duration was calculated by reviewing all available peripheral blood counts preceding and following the follow-up bone marrow biopsy to establish the time period during which basophilia remained sustained with no interruption.

**Figure 1:**
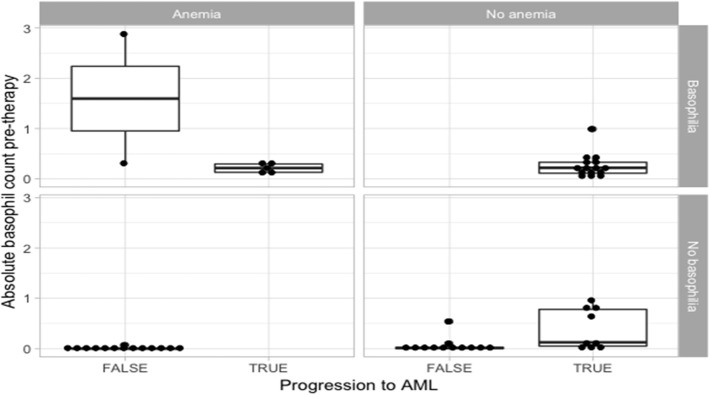
Statistical analysis for the correlation of anemia and basophilia for the evolution of primary myelofibrosis towards acute myeloid leukemia.

Among the patients that developed AML, we report statistically significant differences in the duration of basophilia (*p* = 0.0496, *n* = 26), between patients presenting with pre-myelofibrosis bone marrow fibrosis and the ones that did not, linking a possible common cause of fibrosis and of basophilia. Furthermore, among the patients that developed AML, the duration of basophilia was different between the groups of anemic vs. non-anemic patients (*p* = 0.0649, *n* = 26), but did not quite reach statistical significance. A bigger cohort will be necessary to confirm these observations (Figure 1).

All AML patients had an initial diagnosis of primary myelofibrosis in the fibrotic stage. The diagnosis was reviewed and confirmed at our institution. When the pre-basophilia bone marrow biopsy was available for review at the time of this study, the morphological features were typical for primary myelofibrosis, including the presence of myelofibrotic marrow (MF-2 or MF-3) with increased cellularity and increased granulopoiesis and megakaryopoiesis. Megakaryocytes were predominantly large and pleomorphic with evidence of defective maturation and abnormal chromatin clumping. They were often arranged in dense clusters of several cells and bare megakaryocytic nuclei were also frequently seen. Furthermore, all cases were investigated for *JAK2* V617F, CALR, MPL, and exon 12 mutational status. There was no cytogenetic evolution or change in *JAK2* status associated with the development of basophilia, but we report statistical significance for the CALR status (*p* = 0.00679). Among the AML-negative control group, all patients were investigated for cytogenetic changes. The vast majority of patients with myelofibrosis but without AML were found to be positive in their *JAK2* V617F mutation status (81%). Comparing the cohort with subsequent AML development to the one without AML, the follow-up analysis did not show any statistically significant differences between the groups. In the univariate analysis, patients who progressed to AML had frequently more basophilia, a higher basophilia duration, a higher pre-therapy absolute and relative basophil count and frequently presented more CALR mutations.

Absolute basophil count (ABC) was determined using an automated cell counter and recorded in addition to other hematological and clinical parameters, such as WBC; absolute neutrophil, lymphocyte, monocyte, and eosinophil counts; circulatory blasts; hemoglobin level; platelets; RDW; C reactive protein (CRP); LDH; age; gender; transfusional dependency; constitutional symptoms; blast phase disease; and JAK2, CALR, or MPL mutational status. Spleen and liver size were assessed by palpation. Disease was staged according to the DIPSS prognostic scoring system. ABC of PMF patients was compared to that of 32 age- and gender-matched healthy controls. ABC was significantly higher in PMF patients than in healthy controls (median 0.15 × 10^9^/L vs. 0 × 10^9^/L, *p* < 0.001), with 81% of PMF patients exceeding the upper limit of the ABC reference range of 0.06 × 10^9^/L used in our laboratory. Using the ROC curve analysis, the optimal ABC cut-off value for the purpose of survival analyses was set at 0.1 × 10^9^/L, and patients were separated into a “High ABC” group (>0.1 × 10^9^/L) and a “Low ABC” group ( ≤ 0.1 × 10^9^/L). Patients with high ABC had higher WBC (*p* < 0.001) and LDH (*p* < 0.001) and more frequently had circulatory blasts (*p* < 0.001), constitutional symptoms (*p* = 0.030), and massive splenomegaly (*p* = 0.014). We also observed that ABC as a continuous variable was correlated not only with WBC (*p* < 0.001) and percentage of circulatory blasts (*p* < 0.001) but also with the other components of differential blood count: absolute neutrophil count (*p* < 0.001) and absolute eosinophil count (*p* < 0.001). ABC is correlated with the median time to AML development of 18 months (*p* < 0.01).

Patients that did not progress to AML had more frequently a diagnosis of anemic syndrome (Table 1 and Figure 1). Because the statistically significant variables from the univariate analysis had a high degree of separation between both groups, a multivariate logistic regression was considered not feasible and we chose to grade the importance of the statistically significant variables using a random forest. In the random forest, the variable with the highest mean decrease was ABC pretherapy (10.06) followed by the presence of basophilia (5.37) and anemia (2.51), with an out of bag (OOB) estimate error rate of 14.04%. Duration of basophilia was not included in the model because it is highly correlated with basophilia. Relative basophil count was not included in the model because of its high correlation with the absolute basophil count.

**Table 1:** Detailed statistical analysis for the correlation of anemia and basophilia for the evolution of primary myelofibrosis towards acute myeloid leukemia.

		**Progressed**		
		**No**	**Yes**	***p***
		***n* = 32**	***n* = 32**	
Sex	Female	11 (34%)	13 (41%)	0.796
	Male	21 (66%)	19 (59%)	
Age (quartile 1, quartile 3)		66 (56, 71)	56 (44, 67)	0.0772
Basophilia	No	30 (94%)	9 (31%)	** <0.0001**
	Yes	2 (6%)	20 (69%)	
Basophilia duration (quartile 1, quartile 3) (months)		0 (0, 0)	0 (0, 5.75)	**0.000346**
Follow-up (quartile 1, quartile 3) (months)		33 (4, 56)	23 (2, 40)	0.483
Pre-therapy absolute basophil count (quartile 1, quartile 3) (*10^3^μL)		0.005 (0.000, 0.020)	0.2175 (0.1087, 0.3482)	** <0.0001**
Pre-therapy relative basophil count (quartile 1, quartile 3)		0.00 (0.00, 0.25)	2.00 (1.00, 3.00)	** <0.0001**
Anemia	No	13 (41%)	27 (84%)	**0.0006**
	Yes	19 (59%)	5 (16%)	
Premyelofibrosis bone marrow fibrosis	No	18 (86%)	18 (60%)	0.0641
	Yes	3 (14%)	12 (40%)	
Myelofibrosis grade	1	4 (31%)	2 (20%)	0.642
	2	4 (31%)	2 (20%)	
	3	5 (38%)	6 (60%)	
JAK2	No	3 (19%)	8 (50%)	0.135
	Yes	13 (81%)	8 (50%)	
CALR	No	16 (100%)	9 (56%)	**0.00679**
	Yes	0 (0%)	7 (44%)	

*The Bold Values represent the statistically signifficant values*.

## Discussions

Basophils are typically myeloid cells less frequently seen in a peripheral blood smear, developing in the bone marrow from myeloid progenitors. Their numerous dark azurophilic granules easily distinguish them. The prognosis of myelofibrosis is presently based on clinical characteristics and blood counts at the time of diagnosis. When a malignancy is suspected in the setting of basophilia, the pathologist should contact the attending hematologist. Recommendations should be clearly communicated and should reflect the peripheral smear findings.

The team of Huang et al. have shown that when assessing the risk factors for a myelofibrosis case to evolve clinically into an AML, a peripheral blood blast count of at least 3% or a platelet count of <100 × 10^9^/L are both strong and independent predicting factors for a leukemic transformation in MPNs (9). Morel et al. have shown on behalf of the International Working Group for Myeloproliferative Neoplasms Research and Treatment, on 525 PMF that a leukocytosis of (>25 × 10^9^) predicts poor survival (10). Our data show that patients with PMFs that develop persistent and significant basophilia (at least 3 months) during their disease have an increased risk of later developing AML. When analyzing the statistically significant differences in duration of basophilia among patients that did vs. did not present pre-myelofibrosis bone marrow fibrosis, a possible explanation might be that the action of TGF-β1 secreted by myelofibrosis megakaryocytes acts on both bone marrow fibroblasts as well as on the common basophilic-eosinophilic precursor (11, 12).

**Figure 2:**
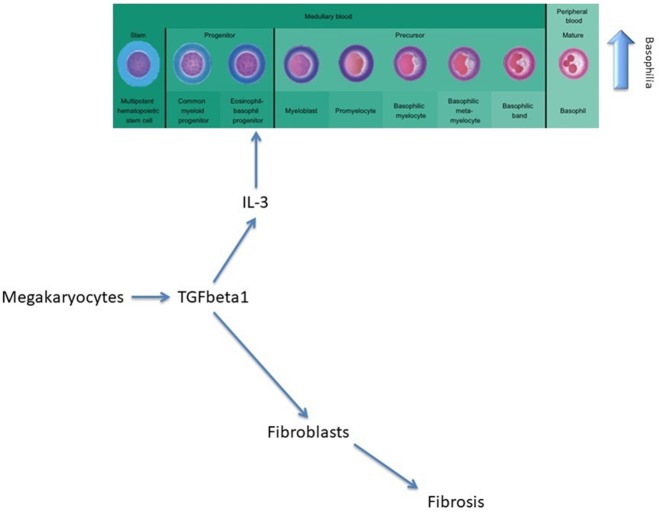
The role of TGF-beta in modulating basophilia for primary myelofibrosis that evolvs towards acute myeloid leukemia.

Furthermore, AML development is not influenced by any type of treatment. Basophilia persists even after initiation of therapy and is associated with worsening disease with the presence of anemia, lower platelet counts, transfusion dependency, and the presence of splenomegaly. In PMF, megakaryocytes secrete TGF-β, a cytokine that will afterwards stimulate the secretion of IL-3 and determine the differentiation and proliferation of the eosinophil-basophil progenitor, thus causing basophilia, and subsequent fibrosis (Figure 2) (11).

**Figure 3:**
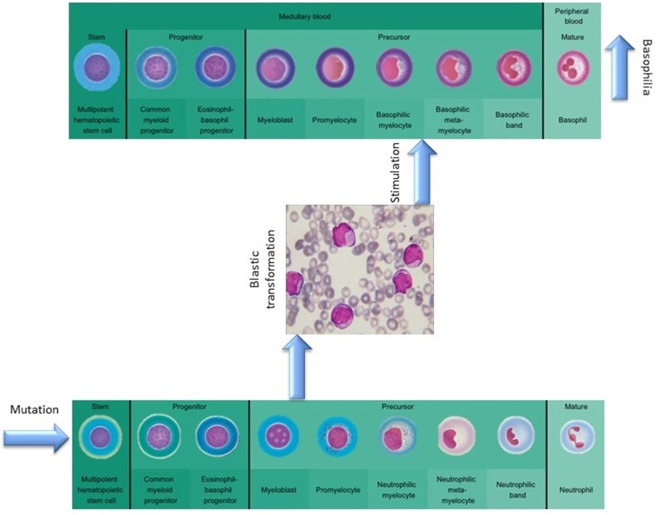
The role of mutational background in modulating basophilia for primary myelofibrosis that evolvs towards acute myeloid leukemia.

**Figure 4:**
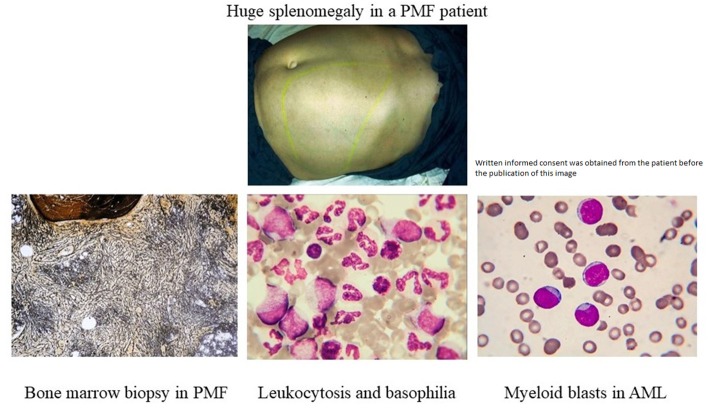
Clinical evolution of a patient with basophilia towards acute myeloid leukemia.

The absence of cytogenetic changes in the clonal evolution of the disease supports the hypothesis that the granulocyte expansion is most likely linked to clonal heterogeneity, as a new clone might have risen during disease progression (Figure 3). The malignant myeloid progenitors in AML secrete TGF-β and thus stimulate the differentiation and proliferation of the common eosinophil-basophil progenitor, thus causing both progressive eosinophilia and basophilia (13, 14), as also shown by Boiocchi et al. (8) (Figure 3). Ohmori et al. found that the IL-3 receptor expression is upregulated in the basophil progenitors, but not in the eosinophil lineage progenitors, and that adding IL-3 complex results in a 4-fold increase in the basophil/mast cell progenitors in the spleen (15). This might explain splenomegaly in PMF patients that evolve toward AML, as depicted in Figure 4. The hypothesis of clonal heterogeneity in PMF is gaining increasing support as it was shown to be the main mechanism responsible for two different types of leukemic trans-formation seen in JAK2 mutation-positive myeloproliferative neoplasms, one with JAK2 mutation-positive blasts and one with JAK2 wild-type blasts. (16). Clonal heterogeneity was also shown in MPNs with del(20q) and myelodysplastic syndromes (MDS) with isolated del(5q) and positive JAK2 V617F mutation (17, 18). Most importantly, the clonal heterogeneity hypothesis, stating that a MPN might have a so-called “accelerated phase” and turn into an AML was shown by Boiocchi et al. (8), who have shown that MPNs with monocytosis have an increased risk of later developing AML.

Both essential thrombocythemia and pre-fibrotic myelofibrosis are WHO-defined entities that may evolve toward a primary myelofibrosis with a high DIPSS risk score or acute leukemia. In PMF, the *CALR* mutation is less studied in comparison to the *JAK2* mutation. Our study showed no cytogenetic evolution or change in JAK2 status associated with the subsequent development of basophilia, but it did show statistical significance regarding *CALR* status (*p* < 0.05). Still, the Italian Collaborative Group for Myeloproliferative Disorders showed that when analyzing 404 patients with either ET or pre-fibrotic PMF, *CALR* mutations were more frequent in pre-fibrotic PMF than in ET (35·8 vs. 17·8%, *P* < 001). Pre-fibrotic PMF patients had shorter overall survival (*P* < 001) and a trend toward higher incidence of leukemic evolution when compared to ET patients (19). Still, the mutational status of CALR must be studied on larger cohorts before a clear statement is made.

## Conclusion

The presented research confirms, at least partially, the previous data on the existence of an “accelerated phase” in myelofibrosis progression and emphasizes the role of basophils in the pathogenesis of PMF. Basophilia is easy to follow, a useful dynamic prognostic parameter and a good indicator for disease progression, should the blast count of cytogenetics remain unchanged. Still, additional data are needed to confirm these findings. Presently, we can still state that because of the rapid disease progression after basophilia appears, the groups of myelofibrosis patients should be monitored separately in a more accurate manner, as they might be more likely to progress to AML. The manuscript presents at this point a hypothesis and theory, proven in a single-center population, and should be regarded as such. The main limitation of the manuscript is the small number of patients enrolled, and the research must be further confirmed by large multi-center cohorts.

## Data Availability

All datasets generated for this study are included in the manuscript/supplementary files.

## Author Contributions

JD and PT wrote the manuscript. SP did the statistical analysis. CT and DD supervised the work. All authors contributed to data gathering.

### Conflict of Interest Statement

The authors declare that the research was conducted in the absence of any commercial or financial relationships that could be construed as a potential conflict of interest.

## References

[1] Mitre E Nutman TB. Basophils, basophilia, and helminth infections. Chem Immunol Allergy. (2006)90:141–56. 10.1159/000088886 16210908

[2] Kane JP. Infectious basophilia? Am J Hematol. (2016) 91:E8. 10.1002/ajh.24243 26572775

[3] Tomuleasa C Selicean S Gafencu G Petrushev B Pop L Berce C. Fibroblast dynamics as an *in vitro* screening platform for anti-fibrotic drugs in primary myelofibrosis. J Cell Physiol. (2018) 233:422–33. 10.1002/jcp.25902 28294327

[4] Barbui T Thiele J Gisslinger H Kvasnicka HM Vannucchi AM Guglielmelli P. The 2016 WHO classification and diagnostic criteria for myeloproliferative neoplasms: document summary and in-depth discussion. Blood Cancer J. (2018) 8:15. 10.1038/s41408-018-0054-y 29426921 PMC5807384

[5] Valent P Sotlar K Blatt K Hartmann K Reiter A Sadovnik I. Proposed diagnostic criteria and classification of basophilic leukemias and related disorders. Leukemia. (2017) 31:788–97. 10.1038/leu.2017.15 28090091 PMC7115817

[6] Visconte V Shetty S Przychodzen B Hirsch C Bodo J Maciejewski JP. Clinicopathologic and molecular characterization of myeloid neoplasms with isolated t(6;9)(p23;q34). Int J Lab Hematol. (2017) 39:409–17. 10.1111/ijlh.12641 28318095 PMC8404557

[7] Knill C SU-E-T-50: updating a familiar clinical tool: including structures in gamma index calculations. Med Phys. (2011) 38:3497 10.1118/1.3612001

[8] Boiocchi L Espinal-Witter R Geyer JT Steinhilber J Bonzheim I Knowles DM. Development of monocytosis in patients with primary myelofibrosis indicates an accelerated phase of the disease. Mod Pathol. (2013) 26:204–12. 10.1038/modpathol.2012.165 23018876

[9] Huang J Li CY Mesa RA Wu W Hanson CA Pardanani A. Risk factors for leukemic transformation in patients with primary myelofibrosis. Cancer. (2008) 112:2726–32. 10.1002/cncr.23505 18404742

[10] Morel P Duhamel A Hivert B Stalniekiewicz L Demory JL Dupriez B. Identification during the follow-up of time-dependent prognostic factors for the competing risks of death and blast phase in primary myelofibrosis: a study of 172 patients. Blood. (2010) 115:4350–5. 10.1182/blood-2009-12-255943 20308601

[11] Sillaber C Geissler K Scherrer R Kaltenbrunner R Bettelheim P Lechner K. Type β transforming growth factors promote interleukin-3 (IL-3)-dependent differentiation of human basophils but inhibit IL-3-dependent differentiation of human eosinophils. Blood. (1992) 80:634–41. 1379084

[12] Ciurea SO Merchant D Mahmud N Ishii T Zhao Y Hu W. Pivotal contributions of megakaryocytes to the biology of idiopathic myelofibrosis. Blood. (2007) 110:986–93. 10.1182/blood-2006-12-064626 17473062 PMC1924766

[13] Fortunel NO Hatzfeld A Hatzfeld JA. Transforming growth factor-β: pleiotropic role in the regulation of hematopoiesis. Blood. (2000) 96:2022–36. 10979943

[14] Blobe GC Schiemann WP Lodish HF. Role of transforming growth factor β in human disease. N Engl J Med. (2000) 342:1350–8. 10.1056/NEJM200005043421807 10793168

[15] Ohmori K Luo Y Jia Y Nishida J Wang Z Bunting KD. IL-3 induces basophil expansion *in vivo* by directing granulocyte-monocyte progenitors to differentiate into basophil lineage-restricted progenitors in the bone marrow and by increasing the number of basophil/mast cell progenitors in the spleen. J Immunol. (2009) 182:2835–41. 10.4049/jimmunol.0802870 19234178 PMC2756103

[16] Beer PA Delhommeau F LeCouédic JP Dawson MA Chen E Bareford D. Two routes to leukemic transformation after a JAK2 mutation-positive myeloproliferative neoplasm. Blood. (2010) 115:2891–900. 10.1182/blood-2009-08-236596 20008300

[17] Schaub FX Jäger R Looser R Hao-Shen H Hermouet S Girodon F Clonal analysis of deletions on chromosome 20q and JAK2-V617F in MPD suggests that del20q acts independently and is not one of the predisposing mutations for JAK2-V617F. Blood. (2009) 113:2022–7. 10.1182/blood-2008-07-167056 19047681

[18] Sokol L Caceres G Rocha K Stockero KJ Dewald DW List AF. JAK2V617Fmutation in myelodysplastic syndrome (MDS) with del(5q) arises in genetically discordant clones. Leuk Res. (2010) 34:821–3. 10.1016/j.leukres.2009.09.016 19819015

[19] Rumi E Boveri E Bellini M Pietra D Ferretti VV Sant'Antonio E. Clinical course and outcome of essential thrombocythemia and prefibrotic myelofibrosisaccording to the revised WHO 2016 diagnostic criteria. Oncotarget. (2017) 8:101735–44. 10.18632/oncotarget.21594 29254200 PMC5731910

